# Factors Associated with the Discordance between Perception of Being HIV Infected and HIV Sexual Risk Taking among Social Media–Using Black, Hispanic, and White Young Men Who Have Sex with Men

**DOI:** 10.1177/2325958220919260

**Published:** 2020-04-21

**Authors:** Christopher L. Bennett, Sarah J. Marks, Joshua G. Rosenberger, José A. Bauermeister, Melissa A. Clark, Tao Liu, Kenneth H. Mayer, Roland C. Merchant

**Affiliations:** 1 Department of Emergency Medicine, Brigham and Women’s Hospital, Harvard Medical School, Boston, MA, USA; 2 Department of Biobehavioral Health, Pennsylvania State University, University Park, PA, USA; 3 School of Nursing, University of Pennsylvania, Philadelphia, PA, USA; 4 Department of Health Services, Policy and Practice, School of Public Health, Brown University, Providence, RI, USA; 5 Department of Biostatistics, Center for Statistical Sciences, School of Public Health, Brown University, Providence, RI, USA; 6 Fenway Health and Division of Infectious Diseases, Department of Medicine, Beth Israel Deaconess Medical Center, Harvard Medical School, Boston, MA, USA

**Keywords:** HIV/AIDS prevention education, community-based participatory evaluation, research, HIV/AIDS transmission &/or risk, condom use, HIV/AIDS knowledge, HIV/AIDS transmission, HIV-related risk behaviors, sexual risk-taking

## Abstract

Among HIV-uninfected, social media–using black, Hispanic, and white young men who have sex with men (YMSM) who had condomless anal sex but had not been HIV tested within the past year, we aimed to determine the extent of discordance between perception of having an undiagnosed HIV infection and HIV risk-taking behaviors. Despite reporting condomless anal sex without HIV testing, 64% of 358 YMSM participants perceived having an undiagnosed HIV infection as “unlikely” and 12% as “impossible.” Having a primary care provider and being Hispanic were associated with greater discordance. Interventions to decrease the discordance between perceived and actual HIV risk are needed for this higher HIV risk population.

**What Do We Already Know about This Topic?**

Discordance between self-perception of low risk for HIV infection and actual HIV sexual risk taking is not well understood and may contribute to the ongoing HIV epidemic.

**How Does Your Research Contribute to the Field?**

In a national sample of a high-risk population of individuals who perceive themselves to be at low risk for an undiagnosed HIV infection but engage in HIV sexual risk-taking behaviors, we found greater discordance among that those with established primary care and those who identify as Hispanic.

**What Are Your Research’s Implications toward Theory, Practice, or Policy?**

Our study is a step forward and (1) supports the existence of discordance between perceived risk and actual risk-taking behavior, (2) identifies a high-risk population that suggests culturally tailored interventions are required, and (3) demonstrates a need for primary care providers to screen patients for HIV testing based on specific risk-taking behaviors and not rely of self-perceptions of risk.

## Introduction

Discordance between self-perceived low risk for having an undiagnosed HIV infection and actual HIV sexual risk taking has been observed previously.^1-3^ However, the reasons for the dissociation have not been well understood, and findings from previous studies conflict.^4-7^ Lower risk perception can translate into decreased utilization of HIV testing which, in turn, may lead to a reservoir of individuals with low-risk perception but with undiagnosed HIV infections. A better understanding of the discordance between risk perception and risk-taking behaviors is needed given that (1) self-perceived risk is considered a vital factor in motivating HIV testing and avoiding HIV risk and (2) those unaware of their HIV infections contribute to a sizable proportion of ongoing HIV transmission. Further, given that HIV in the United States disproportionately affects young adult men who have sex with men (YMSM), particularly men from racial and ethnic minoroties,^8,9^ further study is needed to clarify whether there are particular populations in which this risk discordance is more pronounced.

Previous works examining the discordance between risk perception and sexual risk-taking behaviors have been geographically limited to regional instead of national studies or have not focused on populations known to have higher rates of HIV infection. National studies focused on these populations could determine the extent of discordance between perception of having an undiagnosed HIV infection and self-reported HIV sexual risk taking among YMSM and help better understand potential reasons for this discordance. Findings from such work might assist in the development of interventions to reduce discordance, improve uptake in HIV testing, and lead to decreases in HIV transmission. To better understand the extent of this discordance, we recruited a national sample of black, Hispanic, and white YMSM for a study on HIV testing. In this secondary analysis, we focused on YMSM who had condomless anal intercourse (CAI) within the past year yet had not been tested for HIV within the past year (or never had been tested) to assess extent of discordance between self-perceived risk of an undiagnosed HIV infection as it relates to sexual risk-taking behaviors.

## Methods

This study aimed to identify HIV risk-taking behaviors and demographic characteristics that might explain the discordance between self-perception of an undiagnosed HIV infection and HIV sexual risk-taking behaviors. This investigation was a secondary analysis of data from a larger study on HIV testing in YMSM that recruited 18- to 24-year-old HIV-uninfected black, Hispanic, and white YMSM from across the United States using multiple social media platforms between August and December 2014. As described previously, these platforms included Bender, BlackGayChat, Facebook, Grindr, Growlr, Manhunt, OkCupid, Pinterest, and Reddit.^10^ Young men who have sex with men participants were study eligible if they reported prior anal intercourse and had never had a positive HIV test. Participants completed a 10-minute anonymous online survey about HIV testing practices, HIV sexual risk-taking behaviors, and perception of having an undiagnosed HIV infection. Respondents’ self-perception of being infected with HIV was measured on a 5-point numerical and word scale (0, not possible at all; 1, not likely; 2, somewhat likely; 3, likely; or 4, very likely). As an incentive for study participation, respondents were offered entry in a lottery for a limited number of US$100 gift cards. For this secondary analysis, we included only YMSM who reported CAI within the prior year with casual or exchange partners yet had not been tested for HIV either within the prior year or had never been tested. Casual partners were defined as partners that respondents had sex with but didn’t feel committed. Exchange partners were defined as transactional partners that respondents gave money, drugs, or other things to pay for sex. To focus solely on HIV sexual risk taking, we excluded 12 participants who reported injection drug use.

The question assessing perception of an undiagnosed HIV infection was: “How likely is it that you are infected with HIV, but might not know it?” Responses to this question were collapsed into 3 categories: “not possible,” “not likely,” and “likely.” The “likely” category was a combination of the responses “somewhat likely,” “likely,” or “very likely” to reflect the concept of perceiving a possibility of having an undiagnosed HIV infection. Discordance between perception of having an undiagnosed HIV infection and reported HIV sexual risk-taking behaviors was examined by comparing responses to the HIV sexual risk-taking questions against undiagnosed HIV infection categories (“likely,” “not likely,” and “not possible”). Participants who indicated that an undiagnosed HIV infection was either “not likely” or “not possible,” despite having concurrently reported sexual risk-taking behaviors were deemed discordant.

Proportions for categorical variables were compared using Fisher exact tests, χ^2^ tests, and Kruskal-Wallis tests. Demographic characteristics associated with discordance were identified by measuring the strength of their association with perceptions about the possibility of having an undiagnosed HIV infection. Multinomial logistic regression models were constructed because the proportional odds assumption was not satisfied for ordinal models. Odds ratios with corresponding 95% confidence intervals were estimated; α = .05 was used for statistical significance. Stata version 14.2 (Stat Corp, College Station, Texas) was used for statistical analysis.

### Ethical Approval and Informed Consent

Institutional review board approval was granted for this study at Brown University, and all participants gave informed consent. This research was supported by a grant from the National Institute of Nursing Research (R21 NR023869).

## Results

### Participant Characteristics

The original sample reflected 2275 YMSM aged 18 to 24 years; from this there were 358 YMSM who reported having CAI within the prior year with casual or exchange partners, had not been tested for HIV within the prior year or never had been tested, and denied injection drug use. Among the 358 YMSM, only 4 reported having taken pre-exposure prophylaxis (PrEP). The participants’ median age was 22 years. Most were white or Hispanic; the majority had a college education and lived in an urban or suburban area. Most had a primary care provider (PCP) and had health-care insurance (Table 1). Demographic characteristics were similar according to perception of having an undiagnosed HIV infection except that having a PCP was less frequent among those who thought an undiagnosed infection was “likely” (compared to those who thought it was “not likely” or “not possible”). Hispanic YMSM were the greater race/ethnic group among those who thought an undiagnosed HIV infection was “not possible” (Table 1).

**Table 1.** Demographic Characteristics of Participants Who Have Had Condomless Anal Intercourse (CAI) Within the Past Year and Who Have Not Been Tested within the Past Year by Perception of an Undiagnosed HIV Infection among Participants Who Have Ever Had Casual or Exchange Sex.

	Perception of an Undiagnosed HIV Infection				
	Overall	Likely, n = 86	Not Likely, n = 230	No Possibility, n = 42	*P* Value
Age, median (IQR)	22 (20-23)	21 (20-23)	22 (20-23)	21 (19-23)	.49
	%	%	%	%	
Race/Ethnicity					.03
Black	15	16	14	17	
White	42	40	47	21	
Hispanic	42	44	38	62	
Education completed					.12
Less than high school diploma/GED	8	13	6	12	
High school diploma/GED	16	17	14	26	
Some college	61	57	65	50	
Bachelor’s degree or more	14	11	15	12	
Did not know/refused to answer	0.3		0.4		
Community size					.49
Large city or surrounding suburb	31	30	30	38	
Medium city or surrounding suburb	32	33	35	19	
Small city	15	14	13	24	
Town	15	16	15	17	
Rural area	6	7	6	2	
Did not know/refused to answer	1		1		
Has PCP or Clinic					.02
Yes	59	48	63	62	
No	38	51	34	36	
Did not know/refused to answer	2	1	3	2	
Has insurance					.17
Yes	73	69	76	64	
No	24	28	21	33	
Did not know/refused to answer	3	3	3	2	

Abbreviations: IQR, interquartile range; GED, general equivalency diploma; PCP, primary care provider.

### Discordance between Perception of Undiagnosed HIV Infection and Self-Reported HIV Sexual Risk Taking

Among the 358 participants, almost all reported having CAI with casual partners, while approximately one-fifth reported CAI with exchange partners; nearly half reported CAI within the prior month (Table 2). Of the participants who reported that their risk for HIV was “not likely” or “not possible,” nearly all reported having CAI with casual partners, with almost 30% of the “no possibility group” reporting CAI with exchange partners. The majority (84% of “not likely” and 81% of “not possible”) reported CAI within the prior 6 months, and almost all had multiple casual or exchange CAI partners.

**Table 2.** Reported HIV Sexual Risk-Taking Behaviors by Perception of an Undiagnosed HIV Infection.

		Perception of an Undiagnosed HIV Infection			
	Overall	Likely, n = 86	Not Likely, n = 230	No Possibility, n = 42	*P* Value
CAI with casual partners (%)	99	100^a^	100^b^	98	.29
CAI with exchange partners (%)	21	22^c^	19^d^	29	.33
Last CAI (%)					
Less than 1 month	49	57	47	43	.51
Between 1 and 6 months	35	30	37	38	
Between 6 months to 1 year	16	13	17	20	
Median number of casual partners (IQR) among participants who had at least one casual partner^e^	4 (2-10)	6 (3-16)	4 (2-8)	5 (2-10)	.10
Median number of exchange partners (IQR) among participants who had at least one exchange partner^f^	3 (2-7)	6.5 (2-35)	3 (2-6)	2 (1.5-6.5)	.50

Abbreviations: CAI, condomless anal intercourse; IQR, interquartile range.

^a^ Two participants did not know/refused to respond.

^b^ Two participants did not know/refused to respond.

^c^ Three participants did not know/refused to respond.

^d^ One participant did not know/refused to respond.

^e^ Partners based on number reported top partners and bottom partners; actual number of partners may be lower.

^f^ Partners based on number reported top partners and bottom partners; actual number of partners may be lower.

### Demographic Characteristics Associated with Discordance between Perception of Undiagnosed HIV Infection and Self-Reported HIV Sexual Risk Taking

In the multinomial logistic model of the entire study sample, having a PCP was associated with perceiving undiagnosed HIV infection as “not likely” or “not possible” when compared to “likely” among YMSM who reported CAI yet had never been tested or not been tested for HIV within the prior year (Table 3). Hispanics were more likely to perceive no possibility of infection (compared to “likely infected”). No other race or ethnicity differences were observed for perceptions of possible infection.

**Table 3.** Multinomial Multivariate Logistic Model of Factors Associated with Having No Self-Perceived Perception of HIV Infection or Not Likely Infected among Participants Who Have Ever had Either Casual or Exchange Condomless Anal Intercourse (CAI) and Have Not Been Tested in the Past Year (n = 341).

	Self-Perception of Not Likely Infected Versus Likely Infected, OR (95% CI)	Self-Perception of No Possibility of Infection Versus Likely Infected, OR (95% CI)
Age	1.01 (0.94-1.24)	1.01 (0.83-1.23)
Race/ethnicity		
Black versus white	0.63 (0.29-1.37)	1.79 (0.51-6.27)
Hispanic versus white	0.73 (0.41-1.30)	2.68 (1.05-6.79)
Large city versus medium city and smaller	1.15 (0.64-2.04)	1.42 (0.61-3.29)
Has insurance versus no insurance	1.03 (0.53-2.02)	0.54 (0.20-1.47)
Has PCP versus no PCP	1.82 (1.001-3.30)	2.89 (1.09-7.63)
Education completed/enrolled in HS or college versus not currently enrolled in high school/college and not completed college	0.47 (0.20-1.11)	1.12 (0.54-2.30)

Abbreviations: HS, high school; OR, odds ratio; CI, confidence interval; PCP, primary care provider.

## Discussion

The findings here support the existence of discordance between perception of having an undiagnosed HIV infection and HIV sexual risk-taking behaviors among higher HIV risk YMSM. All YMSM in this secondary analysis reported engaging in CAI with casual or exchange partners (in the absence of HIV testing in the prior year or previously), yet 64% perceived that an undiagnosed HIV infection was “not likely” and 12% that it was “not possible.” Of the YMSM who had CAI within the prior year, had not been HIV tested within the last year or previously, and perceived themselves as “not likely” or “not possible” of having an undiagnosed HIV infection, the majority reported CAI with casual partners and most had CAI within the prior 6 months. If perception of an undiagnosed HIV infection was concordant with reported HIV risk taking, the proportion engaging in CAI with casual and exchange partners should be much lower. HIV PrEP cannot account for the discordance given that only 1% reported ever using PrEP, which is important given its efficacy for prevention of HIV acquisition after condomless sex.^11^

Hispanic YMSM were more likely to perceive no possibility of infection, despite self-reported HIV risk-taking behaviors, and having a PCP was associated with lower perception of having an undiagnosed HIV infection in the study sample. This observation underscores US Centers for Disease Control and Prevention (CDC) data demonstrating that Hispanic/Latino individuals accounted for 26% of all new HIV diagnoses in 2016 and that 1 in 6 Hispanic/Latino individuals were estimated to be unaware of their HIV infection.^9^ The findings in this study are consistent with previous work demonstrating lower HIV/AIDS knowledge among Hispanic adolescents.^12^ This identified discordance also exists in the context of language barriers and socioeconomic challenges disproportionately faced by this population that may either (1) decrease likelihood of seeking testing or (2) make it more difficult for these individuals to be tested. Although beyond the scope of this current study (given that we did not query immigration status), it is possible that our findings may also stem from fear of discrimination surrounding disclosure of immigration status. Regardless, our results suggest that culturally tailored educational interventions for Hispanic/Latino YMSM are needed to reduce the ongoing discordance between perception of having an undiagnosed infection and HIV risk-taking behavior. Given the potential impact of such efforts, initiatives by the CDC^9^ and other national funding bodies could supplement current efforts with both English- and Spanish-based initiatives targeted to this population.

We were surprised to find that having a PCP was associated with discordance between perception of an undiagnosed HIV infection and risk behavior. It would be expected that having a PCP could decrease the likelihood of HIV transmission by encouraging condom usage, providing PrEP, explaining the role of CAI in HIV transmission, and conducting CDC-recommended HIV testing. One possible explanation is that having a PCP may have falsely conferred a reduced perception of having an undiagnosed HIV infection, potentially reflecting an optimistic bias (eg, “I must be free from HIV since I am being cared for by my doctor, who would test me for it if (s)he thought I had it”). Also, the provider–patient relationship could be complicated if YMSM do not feel comfortable disclosing their risk behavior. Regardless of the explanation, this finding should be alarming to clinicians who may rely on patients’ reports of their HIV risk when considering HIV testing. Instead, clinicians should query all patients on specific risk behaviors in a manner that encourages disclosure and allows for patient education. Primary care providers (and other care providers) should test based not upon patients’ self-perceived risk but instead upon actual risk-taking behaviors and current CDC testing guidelines. Further, this observation underscores the need for PCPs to conduct recurrent screens for risk-taking behaviors, reduce HIV misconceptions, conduct routine HIV testing, and implement risk-mitigating behaviors, such as condom usage and PrEP (notably among their Hispanic/Latino patients).^1^

Previous studies have documented discordance between perceived HIV risk and sexual risk-taking behaviors, but the reason(s) underlying this discordancy remains unclear; multiple explanations have been suggested, including gaps in HIV/AIDS-related knowledge and anchoring and optimism biases.^13,14^ Regarding knowledge, others have proposed that individuals on both ends of the spectrum of HIV/AIDS-related knowledge (both without any knowledge and with a lot of knowledge) should have lower HIV risk perception.^2^ Without knowledge, individuals cannot perceive HIV/AIDS risk. Likewise, having substantial knowledge about HIV/AIDS prevention and transmission should also result in low perceived risk, resulting from a reduced “fear or dread” of HIV risk, concurrent with a “presumed increased controllability.” Anchoring refers to a cognitive bias wherein incorrect judgements, decisions, and/or risk perceptions may be made, either as a result of anchoring on prior negative HIV testing or as a result of cues in questions attempting to assess risk.^2,15,16^ Optimism refers to a bias of denial and distancing leading to erroneously construed risk estimation. Individuals create an illusion of low HIV risk, out of touch with the reality of their actual risk-taking behaviors.^2,17^ On the other hand, the assumption that sexual behaviors are governed by the same thought processes as other human behaviors might be inherently flawed; sexual risk behaviors and motivators surrounding these interactions and individual’s recall and perceived risk might simply be illogical.^17^ As such, screening for HIV should be independent of risk perceptions and instead be based upon actual sexual risk-taking behaviors. Future research should examine the reasons for discordance between perception of an undiagnosed HIV infection and risk-taking behaviors as a means to overcome discordance and reduce the burden of undiagnosed HIV infections.

We acknowledge several limitations with this work. This investigation is a secondary study, and therefore was limited by the data available for analysis. We also cannot claim that the findings are externally valid to other YMSM. However, our sample includes a geographically diverse subset of YMSM from across the United States. Further, the data collected were self-reported. Objective data (eg, HIV test results) were not verified, although there might be no practical method to do so. Our inability to identify differences among groups compared in the investigation could be due to insufficient sample size, although we cannot confirm this. Moreover, serosorting (the potential for respondents to preferentially avoid engaging in intercourse with HIV-positive partners) and psychosocial factors (eg, denial, dissociation, optimistic bias) were not obtained and therefore could not be investigated as possible explanations for discordance.

In conclusion, this national study of US black, Hispanic, and white YMSM adds to a growing body of literature demonstrating discordance between perceptions of having an undiagnosed HIV infection and higher risk sexual behaviors in multiple populations. It demonstrates a need for further study aimed at better clarifying the causes underlying this discordance and how this discordance might be reduced to increase HIV testing and reduce HIV risk taking among this higher HIV risk population.
